# 

**Published:** 1887-06

**Authors:** 


### EDITORIAL.

### American Dental Association.

What about it? When and where will it be held? These are the questions that have been under consideration for some time, with as usual, a variety of opinions about it. There seems to be a constitutional difficulty in the way of postponement till 1888. This was thought to be the best thing to do, till new light dawned, then the question again arose: When, and where?

Various suggestions have been made. In regard to time, the general opinion is that it should be the week before the meeting of the International Medical Congress; if so, it should be at some point near or on the way to Washington, to save time and expense to the members as far as possible. Deer Park has been suggested; this is a good idea in some respects. The next best thing to this, and perhaps a better would be to go to Old Point Comfort, and at least greet the brethren of the South, if nothing more.

The postponement of the meeting till the latter part of November, and then held in St. Louis, would seem to be in the interest of the Association; there would in all probability be a much larger attendance, and far more effort could be given to it than is possible during the summer. The interests of the Association would be much better served, and the profession derive far more benefit by such postponement. St. Louis has never had the Association, and it would seem on several accounts quite desirable and proper that a meeting of the body be held in that city. Now then, O! Ye Powers that be! what say you? Act according to your best judgement, and there will be a general acquiescence.

### THE DENTAL REGISTER,

A MONTHLY MAGAZINE DEVOTED TO THE INTERESTS OF THE

### DENTAL PROFESSION.

*Terms Reduced to* $2.00 *Per Year. Single Copies*, 25 *Cents.*

### EDITED BY PROF. J. TAFT, D.D.S.

—AND—

### Published by SAM'L A. CROCKER & CO., Ohio Dental and Surgical Depot.

The volume commences in January and closes in December.

The REGISTER being issued monthly is always fresh, therefore Indispensable to the practitioner who desires to keep posted up with the new inventions and improvements which are daily developed. Neither expense nor effort will be spared to give value and variety to its contents. Articles will be illustrated with well executed engravings whenever they will add to the interest or assist to explanation.

Its extensive circulation and frequency of issue make it one of the BEST DENTAL ADVERTISING MEDIUMS in the United States.

All subscriptions must begin with January or July.

Local or special notices, five lines or less, will be inserted for $3 each insertion; over five lines, 50 cts. per line.

All advertising bills payable in advance, or at furthest, after the issue of the first number containing the advertisement. All transient advertisements to be paid for in advance.

☞CONTRIBUTIONS SOLICITED.

Exclusively Editorial Communications should be addressed to the Editor.

The latest number of the REGISTER may be ordered with or without other goods at any time, and all letters should be addressed to

SAM'L A. CROCKER & CO., Ohio Dental and Surgical Depot, Cincinnati, O.

